# Photogenerated hole traps in metal-organic-framework photocatalysts for visible-light-driven hydrogen evolution

**DOI:** 10.1038/s42004-022-00713-4

**Published:** 2022-08-06

**Authors:** Zichao Lian, Zhao Li, Fan Wu, Yueqi Zhong, Yunni Liu, Wenchao Wang, Jiangzhi Zi, Weiwei Yang

**Affiliations:** School of Materials and Chemistry, University of Shanghai for Science and Technology, 200093 Shanghai, P. R. China

**Keywords:** Photocatalysis, Nanoparticles, Metal-organic frameworks, Porous materials

## Abstract

Efficient electron-hole separation and carrier utilization are key factors in photocatalytic systems. Here, we use a metal-organic framework (NH_2_-UiO-66) modified with inner platinum nanoparticles and outer cadmium sulfide (CdS) nanoparticles to construct the ternary composite Pt@NH_2_-UiO-66/CdS, which has a spatially separated, hierarchical structure for enhanced visible-light-driven hydrogen evolution. Relative to pure NH_2_-UiO-66, Pt@NH_2_-UiO-66, and NH_2_-UiO-66/CdS samples, the Pt@NH_2_-UiO-66/CdS composite exhibits much higher hydrogen yields with an apparent quantum efficiency of 40.3% at 400 nm irradiation and stability over the most MOF-based photocatalysts. Transient absorption measurements reveal spatial charge-separation dynamics in the composites. The catalyst’s high activity and durability are attributed to charge separation following an efficient photogenerated hole-transfer band-trap pathway. This work holds promise for enhanced MOF-based photocatalysis using efficient hole-transfer routes.

## Abstract (web summary)

Efficient electron-hole separation and carrier utilization are key factors in photosynthetic systems. Here, the authors achieve efficient charge separation following a photogenerated hole-transfer band-trap pathway in the ternary composite Pt@NH_2_-UiO-66/CdS, resulting in photocatalytic hydrogen evolution with good stability and a quantum efficiency of 40.3% at 400 nm irradiation.

## Introduction

Photocatalytic hydrogen evolution via water splitting is a promising way to mitigate current energy and environmental issues^1–7^. Since Fujishima and Honda first reported solar energy conversion for hydrogen evolution using semiconductor-based photocatalysts, we have witnessed the development of artificial photocatalytic systems for enhanced hydrogen evolution reactions (HER)^8–10^. The primary drawback in using a pure semiconductor was fast recombination of photoinduced carriers, which seriously limited photocatalytic efficiency^11,12^. Meanwhile, co-catalysts, such as Pt nanoparticles (NPs) and RuO_2_, have been alternatives that provide an active center for redox reductions, reduce overpotentials for HER or oxidation reactions, and promote fast separations of photoinduced electrons and holes^13–16^. Thus, complex heterostructures with spatial charge separations via fine control of photoinduced carrier dynamics have been fabricated to improve photocatalytic activity.

Metal-organic frameworks (MOFs) are porous crystalline materials with high specific-surface areas, and tunable structures and functionalities. As alternatives to semiconductor photocatalysts that are receiving much interest in a wide range of applications. Unfortunately, MOFs, such as UiO-66 (Zr)^17,18^, exhibit poor visible-light absorption. The introduction of amino groups into the terephthalic acid ligands of UiO-66 (Zr) broaden light absorption to the visible region, and the presence of amino groups does not affect the structural stability of the UiO-66 host^19^. Photoinduced electron dynamics in MOFs have been well investigated, but the kinetics of photogenerated holes and their effect on photocatalytic activity remain poorly understood. Cadmium sulfide (CdS) has been the preferred visible-light photocatalyst for HER. However, photoinduced corrosion and fast recombination of photoinduced electron−hole pairs have severely restricted improvements in its catalytic activity. Thus, fine-tuning photoinduced carrier dynamics is necessary to suppress corrosion by accumulated holes^3,20–27^.

Here, we synthesized a spatial charge structure for the MOF-based photocatalyst (Pt@NH_2_-UiO-66/CdS). Relative to other MOF-based systems (see Supplementary Table 1), it exhibited the highest visible-light photocatalytic activity for HER, with an apparent quantum yield of 40.3% at 400 nm. Direct observation of the carrier dynamics in Pt@NH_2_-UiO-66/CdS, via transient absorption spectroscopy (TAS), revealed that a unique hole-transfer pathway was responsible for the enhanced and stable HER. The present results could help design spatial, multi-phase, heterostructured photocatalysts with high photocatalytic activity and facile control of photoinduced carrier dynamics.

## Results and discussion

### Characterization of materials

**Fig. 1:**
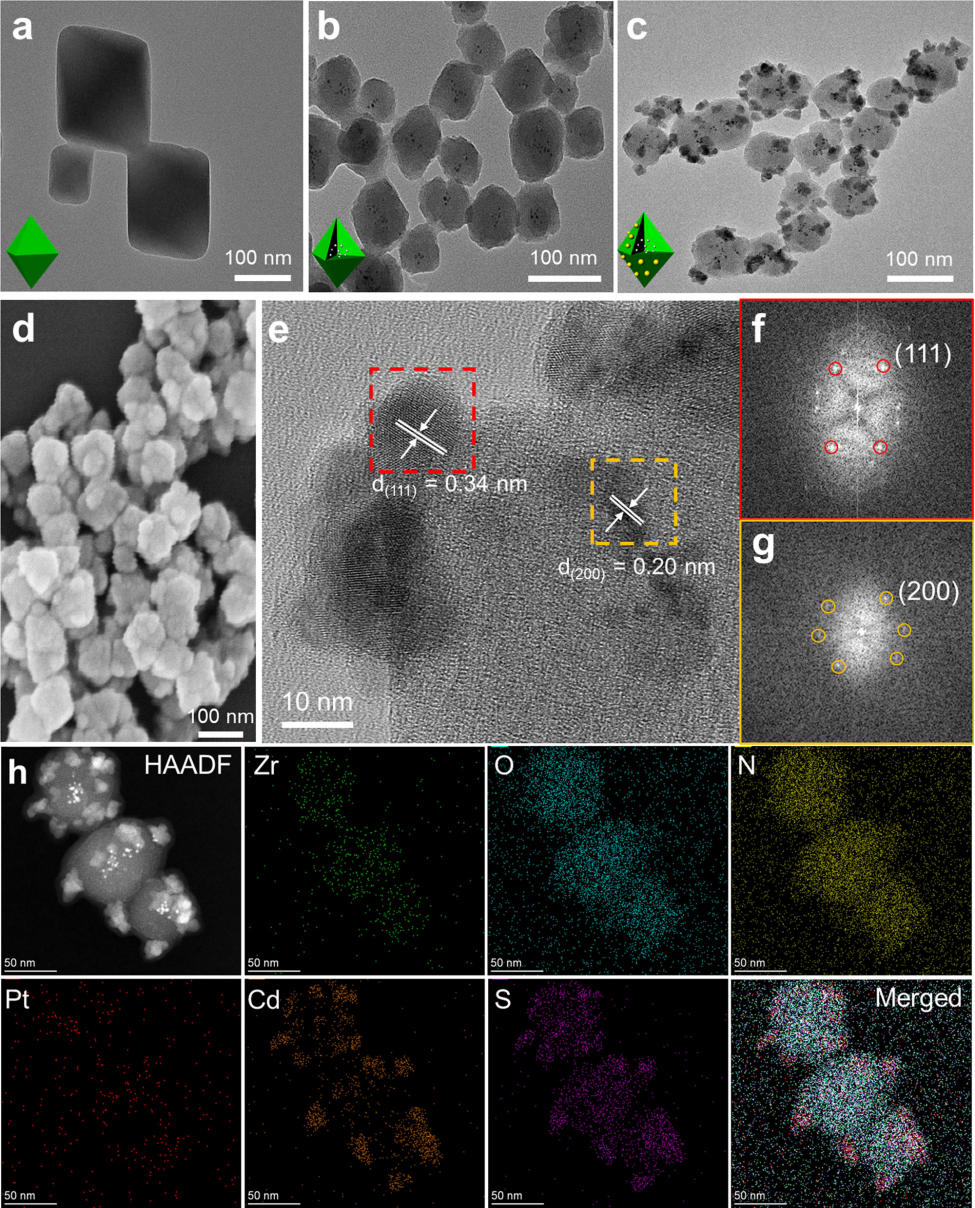
Structural characterization of the nanocrystals. **a**–**c** Representative transmission electron microscopy (TEM) images of (**a**) NH_2_-UiO-66, (**b**) Pt@NH_2_-UiO-66, and (**c**) Pt@NH_2_-UiO-66/CdS, inset: schematic representation (lower left). **d** Scanning electron microscopy image of Pt@NH_2_-UiO-66/CdS. **e** High-resolution TEM image of a single Pt@NH_2_-UiO-66/CdS. **f**, **g** Fast Fourier transform patterns of the CdS phase from <111> direction at the left region of (**e**) and the Pt phase from <001> direction at the right region of (**e)**, respectively. **h**, High-angle annular dark-field (HAADF) scanning TEM and energy-dispersive spectrometry elemental mapping images of Pt@NH_2_-UiO-66/CdS.

We synthesized Pt@NH_2_-UiO-66/CdS heterostructured nanocrystals (HNCs) by in-situ encapsulation of Pt nanoparticles (NPs) into MOFs having regular 100-nm octahedral shapes, and by growing CdS on the outer MOF surface, as shown in Supplementary Fig. 1. Figure 1a shows representative transmission electron microscopy (TEM) images of NH_2_-UiO-66 NCs with regular octahedral structures, which were consistent with scanning electron microscopy images in Supplementary Fig. 2. The Pt NPs (Supplementary Fig. 3) were encapsulated in situ in the MOF shown in Fig. 1b. By growing multiple CdS satellites of 10.5 ± 2.1 nm in size on the outer layer of Pt@NH_2_-UiO-66, Pt@NH_2_-UiO-66/CdS HNCs were formed with a spatial configuration of inner Pt NPs and outer CdS, as shown in Fig. 1c. Figure 1d is a scanning electron microscopy image of Pt@NH_2_-UiO-66/CdS HNCs that shows the CdS NPs attached to the surface of the MOFs. X-ray diffraction patterns in Supplementary Fig. 4 show that the Pt@NH_2_-UiO-66/CdS HNCs were composed of NH_2_-UiO-66 and zinc blend CdS, with Joint Committee on Power Diffraction Standards (JCPDS) no. 10-0454 phases. The Zr/Cd weight ratio was 0.28:14.3, as determined by inductively coupled plasma optical emission spectroscopy (ICP-OES). However, the Pt NPs did not display a diffraction peak due to the low amount of Pt (about 0.023 wt %) determined by ICP-OES (Supplementary Table 2). The high-resolution TEM image in Fig. 1e and Supplementary Fig. 5 revealed the composition of Pt NPs and CdS NPs. The 0.34-nm and 0.20-nm lattice fringes were assigned to zinc blend CdS (111) and Pt (200), respectively, which were consistent with fast Fourier transform patterns [Fig. 1f, g]. High-angle annular dark-field scanning TEM and energy-dispersive spectrometry elemental mapping (Fig. 1h) also verified the formation of ternary heterostructures composed of Pt, NH_2_-UiO-66, and CdS phases.

Furthermore, the Brunauer–Emmett–Teller surface area and pore structures analysis were performed, as shown in Supplementary Fig. 6 and Supplementary Table 3. By comparing the specific-surface area of the NH_2_-UiO-66 (820 m^2^ g^−1^), Pt@NH_2_-UiO-66 (722 m^2^ g^−1^), Pt@NH_2_-UiO-66/CdS (612 m^2^ g^−1^) HNCs, the smaller specific-surface area of Pt@NH_2_-UiO-66/CdS indicated that the CdS occupies the surface sites of the MOF. In addition, compared with that of NH_2_-UiO-66 and Pt@NH_2_-UiO-66, the porous volume of Pt@NH_2_-UiO-66/CdS was increased due to the effects of CdS NPs. We also further verified the composition and interface characteristics of Pt@NH_2_-UiO-66/CdS composites by the X-ray photoelectron spectroscopy (XPS) measurements (Supplementary Fig. 7). First, the XPS spectrum of Supplementary Fig. 7a shows that the Pt@NH_2_-UiO-66/CdS composites contain the elements such as C, N, O, Zr, Pt, Cd, and S, and there are no other impurity elements. Secondly, the binding energies of Cd 3d_3/2_ and 3d_5/2_ of the sample Pt@NH_2_-UiO-66/CdS are around 411.9 and 405.2 eV, respectively, indicating that the Cd in the composite material exists in the +2 valence^28^. In addition, the binding energies of S 2p_1/2_ and 2p_3/2_ are around 162.7 and 161.5 eV, respectively, indicating that S is −2 valence^29^ (Supplementary Fig. 7d, e). Therefore, these XPS results can fully demonstrate the existence of CdS in the Pt@NH_2_-UiO-66/CdS composites. Compared with NH_2_-UiO-66 and Pt@NH_2_-UiO-66, the Zr 3d binding energy of Pt@NH_2_-UiO-66/CdS composite was shifted by 0.1 eV (Supplementary Fig. 7b). It suggested that there was a strong interaction between CdS and NH_2_-UiO-66, rather than a simple physical contact^30^. As shown in Supplementary Fig. 7c, the binding energies of Pt 4f_5/2_ and 4f_7/2_ in the Pt@NH_2_-UiO-66 are around 74.4 and 71.1 eV, respectively, which are assigned to the metallic Pt^31^. However, the binding energies of Pt f_5/2_ and 4f_7/2_ in Pt@NH_2_-UiO-66/CdS were shifted to around 74.7 and 71.3 eV, respectively, indicating that the loading of CdS in the composite caused the binding energy of Pt to be shifted by 0.3 eV, but the valence state of Pt has not changed.

### Optical properties and photocatalytic activity

**Fig. 2:**
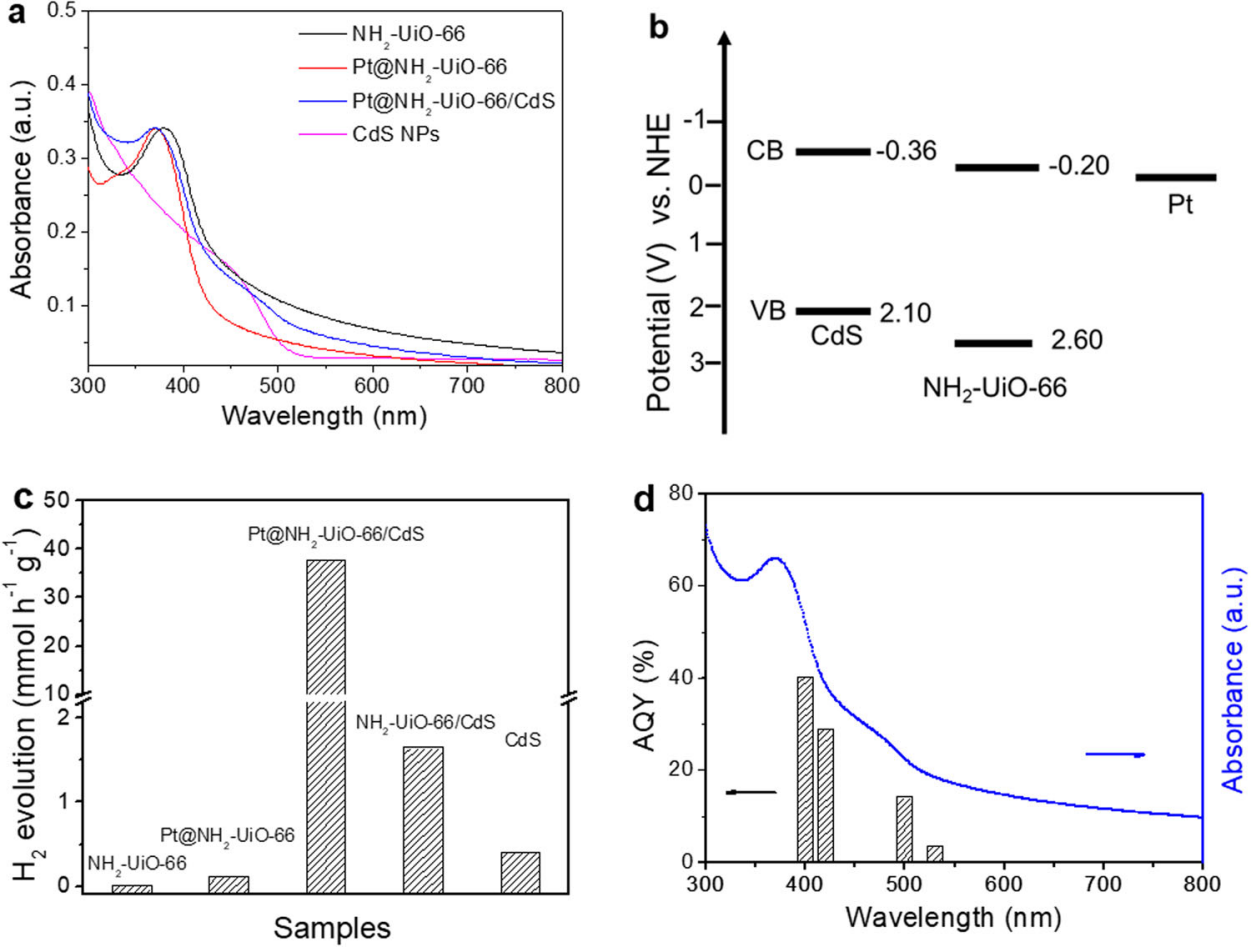
Optical properties, band diagram, and photocatalytic hydrogen evolution. **a** Ultraviolet–visible absorption spectra of NH_2_-UiO-66, Pt@NH_2_-UiO-66, Pt@NH_2_-UiO-66/CdS and CdS nanoparticles (NPs) in dimethyl formamide. **b** Energy levels for Pt, NH_2_-UiO-66, and CdS NPs. The black solid lines represent conduction bands (CBs) and valence bands (VBs) for CdS, NH_2_-UiO-66, and the work function for Pt NPs, respectively. **c** Comparison of the hydrogen evolution rate for NH_2_-UiO-66, Pt@NH_2_-UiO-66, Pt@NH_2_-UiO-66/CdS, NH_2_-UiO-66/CdS and CdS NPs under visible-light (>420 nm), using a lactic acid acetonitrile solution as a sacrificial agent. **d** Apparent quantum yields (AQY) of Pt@NH_2_-UiO-66/CdS at several wavelengths under identical conditions. The blue line shows the absorption spectrum.

The visible-light-driven photocatalytic activity and stability of Pt@NH_2_-UiO-66/CdS HNCs for HER were investigated. Figure 2a shows the ultraviolet-visible absorption spectra of NH_2_-UiO-66, Pt@NH_2_-UiO-66, Pt@NH_2_-UiO-66/CdS, and CdS NPs (also, see Supplementary Fig. 8). Pt@NH_2_-UiO-66/CdS featured the absorption characteristics of MOF at 370 nm and the band-edge absorption of the CdS phase at 500 nm. Figure 2b shows the energy levels of NH_2_-UiO-66 and CdS that were determined in Supplementary Fig. 9; they were consistent with the previous reports^3,32^. As shown in Fig. 2c, the HER visible-light-driven photocatalytic activity (using lactic acid as a sacrificial agent) for Pt@NH_2_-UiO-66/CdS was 37.76 mmol h^−1^ g^−1^, which was higher than that of NH_2_-UiO-66 (0.011 mmol h^−1^ g^−1^), Pt@NH_2_-UiO-66 (0.12 mmol h^−1^ g^−1^), NH_2_-UiO-66/CdS (1.65 mmol h^−1^ g^−1^, Supplementary Fig. 10), and CdS NPs (0.41 mmol h^−1^ g^−1^). The photocatalytic H_2_ evolution has increased with the increase of the amount of catalyst, then decreased when the dosage of catalyst was 10 mg (Supplementary Fig. 11). Thus, the 5 mg of the optimized photocatalyst dosage displays the best catalytic performance. When the photocatalytic H_2_ evolution was normalized by the quality of the catalyst, however, with the increase of the catalyst dosage, the hydrogen evolution rate gradually decreases, possibly due to blocked or scattered light by the excess suspended photocatalysts in the reaction medium^21–23^. To validate the benefits of spatial separation of the co-catalysts in the MOFs, we examined the HER photocatalytic activity for physical mixtures of materials with normalized Pt contents. Supplementary Fig. 12 shows that Pt@NH_2_-UiO-66/CdS exhibited higher activity than the others, which was consistent with better photocatalytic activity of Pt implanted in the MOF^33^. Furthermore, the cascade photoinduced carrier transfer with the spatial charge separation between the ternary phases with strong interactions contributed much to the high activity. The Pt@NH_2_-UiO-66/CdS maintained good stability after seven cycles over twenty-one hours and its morphology had no significant changes, as shown in Supplementary Fig. 13. Various sacrificial agents from small to large molecules were used to reveal the roles in HER photocatalytic activity by consuming photogenerated holes. Supplementary Fig. 14 shows that the lactic acid was the best sacrificial agent, where the carboxyl groups played a more important role than the hydroxyl groups. The large molecules could be oxidized by photogenerated holes in Pt@NH_2_-UiO-66/CdS. In the photocatalytic degradation of macromolecules, the generation of •OH radicals could be detected by fluorescence emission and electron-paramagnetic-resonance spectra. Larger signals were trapped by 5,5-dimethyl-1-pyrroline N-oxide (DMPO)^32^ (see Supplementary Fig. 15), which restricted the HER photocatalytic activity. As shown in Supplementary Fig. 16, with the increase of lactic acid contents (decrease of the pH) in the system, the H_2_ evolution rate of the catalyst gradually increases, which further indicates that the timely removal of holes is beneficial to improve the H_2_ evolution rate. The wavelength-dependent apparent quantum yields (AQYs) of Pt@NH_2_-UiO-66/CdS shown in Fig. 2d reproduced its absorption spectrum, indicating that HER proceeded via photoexcitation. The AQY at 400 nm was 40.3%, which was the highest efficiency relative to previous reports (Supplementary Table 1). These results demonstrated that Pt@NH_2_-UiO-66/CdS had the highest photocatalytic activity and stability, due to the spatial separation of the photoinduced electrons and holes. To reveal the separation and recombination of these charge carriers, we characterized the photoluminescence (PL), transient photocurrent spectra and electrochemical impedance spectroscopy. Pt@NH_2_-UiO-66/CdS exhibited a very low fluorescence intensity relative to that for NH_2_-UiO-66, Pt@NH_2_-UiO-66 and NH_2_-UiO-66/CdS (Supplementary Fig. 17). This suggested greatly inhibited recombination of electron and hole pairs in the HNCs^34^. However, the enhanced photocurrent density of the Pt@NH_2_-UiO-66/CdS promoted transfer kinetics of the photoexcited charge carriers (Supplementary Fig. 17). Meanwhile, the interfacial properties between the electrode and the electrolyte were also investigated using electrochemical impedance spectroscopy measurements. As known, the semicircle at a high frequency in the Nyquist plot may illustrate the charge-transfer process. The diameter of the semicircle is dependent on the charge-transfer resistance, as shown in Supplementary Fig. 17, the Pt@NH_2_-UiO-66/CdS exhibits the smallest arc due to its lowest charge-transfer resistance. Meanwhile, the characteristic frequency in the Bode phase plot of the Pt@NH_2_-UiO-66/CdS film could be estimated a little shift to a lower frequency relative to this of the Pt@NH_2_-UiO-66 film (Supplementary Fig. 17), further indicating that the charge-recombination rate is reduced in the Pt@NH_2_-UiO-66/CdS^35^. These photo-electrochemical properties indicated that Pt@NH_2_-UiO-66/CdS enhanced separation and migration of the photoinduced charge carriers, which was attributed to its unique spatial-phase separation and hierarchical structure.

### Photoinduced carrier dynamics

**Fig. 3:**
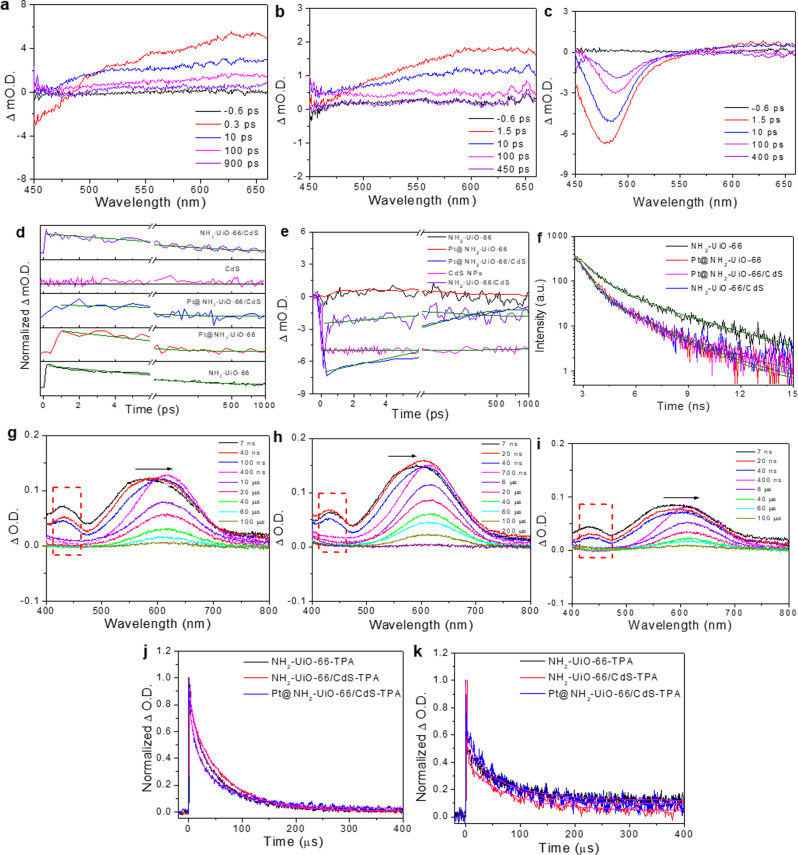
Transient absorption spectra (TAS), kinetic profiles, and time-resolved photoluminescence (PL) spectra. **a**–**c** TAS of **a** NH_2_-UiO-66, (**b**) Pt@NH_2_-UiO-66, and (**c**) Pt@NH_2_-UiO-66/CdS with TA signal, given in optical density (OD) units, upon 400-nm laser excitation. **d**, **e** Kinetic profiles of NH_2_-UiO-66, Pt@NH_2_-UiO-66, Pt@NH_2_-UiO-66/CdS, CdS NPs, and NH_2_-UiO-66/CdS at (**d**) 650 nm, and (**e**) 480 nm, respectively. **f** Time-resolved PL decay profiles for NH_2_-UiO-66, Pt@NH_2_-UiO-66, Pt@NH_2_-UiO-66/CdS and NH_2_-UiO-66/CdS, (400 nm excitation, 450 nm emission). All the olive lines are best fits with a biexponential function. **g**–**i** TAS of (**g**) NH_2_-UiO-66, (**h**) NH_2_-UiO-66/CdS, and (**i**) Pt@NH_2_-UiO-66/CdS with triphenylamine (TPA) in dimethyl formamide (DMF) solution, in the nanosecond to microsecond region. A new species was produced with an absorption at 430 nm upon 355-nm laser excitation. **j**, **k** Kinetic profiles of NH_2_-UiO-66, NH_2_-UiO-66/CdS and Pt@NH_2_-UiO-66/CdS containing TPA DMF solution probing at (**j**) 600 nm to investigate the dynamics of cation radicals (TPA^•+^), and (**k**) 430 nm for the absorption of a photogenerated hole transfer transition band trap to monitor the dynamics of hole transfer, respectively. The best fits using biexponential function are in magenta lines.

Time-resolved transient absorption (TA) measurements were performed to track photoinduced carrier dynamics to investigate electron and hole transfers in the mechanism of photocatalytic HER. Figure 3a shows time-resolved TA spectra of NH_2_-UiO-66 after selective excitation with a 400-nm laser. Band-edge bleaching at less than 450 nm and broad absorption at 600 nm were observed. Fast and slow recovery monitoring at 650 nm is shown in Fig. 3d. Supplementary Table 4 lists the two recovery time constants of 9.8 ps (*τ*_1_) and 225 ps (*τ*_2_), respectively, which may be attributed to electron-trap states in the intermediate band^16,33,36,37^. For Pt@NH_2_-UiO-66, similar features were observed in Fig. 3b, with two decay components of 6.2 ps and 69 ps, respectively. The fast component could be assigned to fast electron transfer from the conduction-band minimum of the MOF to the trap state and then to the Pt sites. The TA characteristics for Pt@NH_2_-UiO-66/CdS shown in Fig. 3c were bleaching at the 480-nm peak and a broad absorption at 600 nm that was weaker than that of CdS NPs and NH_2_-UiO-66/CdS (Supplementary Fig. 18). The first component (29 ps) was the fast-trap process, from possible coupled interactions with the hole transfer from NH_2_-UiO-66 to CdS, and an electron-trap state in NH_2_-UiO-66, where CdS NPs have no hole-trap states. The latter of CdS NPs is indicated by featureless absorption from the TAS and kinetic profiles at 650 nm in the previous report^1,3^. The bleaching at 480 nm for Pt@NH_2_-UiO-66/CdS with the fast-quenching component is shown in Fig. 3e, indicating electron transfers from CdS to NH_2_-UiO-66^38^. This feature was also observed in NH_2_-UiO-66/CdS, with about a 64% decrease in ultrafast quenching (<100 fs), suggesting electron transfers from CdS to NH_2_-UiO-66. The fast recovery with the 9.3-ps time constant indicated that the electron from NH_2_-UiO-66 transferred to the Pt phase. The fs TAS suggested that the electron transferred from CdS to NH_2_-UiO-66, and was then trapped at NH_2_-UiO-66 before finally transferring to the Pt phase. The hole from NH_2_-UiO-66 was transferred to CdS. Time-resolved PL spectroscopy was used to investigate the lifetimes of photoinduced carriers in Pt@NH_2_-UiO-66/CdS^33^. Figure 3f shows the 450-nm PL kinetics for each sample following excitation at the band-edge absorption. The mean PL lifetimes were 1.97 ns, 0.99 ns, 1.05 ns, and 0.95 ns for NH_2_-UiO-66, Pt@NH_2_-UiO-66, NH_2_-UiO-66/CdS and Pt@NH_2_-UiO-66/CdS, respectively (Supplementary Table 5). The shorter PL lifetime of Pt@NH_2_-UiO-66/CdS indicated that the spatial charge-separation of the ternary composites was suppressed by accelerating the radiative recombination of the photoinduced electrons and holes. This strongly indicated that photogenerated carriers transferred in Pt@NH_2_-UiO-66/CdS could have other pathways of nonradiative recombination for high photocatalytic activity.

To understand the hole dynamics in the HER photocatalytic activity, we used triphenylamine (TPA) as a hole indicator. It was oxidized by photogenerated holes to form a cation radical (TPA^•+^) with an absorption feature at 600 nm^39^. Figure 3g shows the μs TAS of NH_2_-UiO-66 upon 355-nm laser excitation in the presence of TPA; the TPA was not excited by the laser (Supplementary Fig. 19). Thus, the decay signal observed at 600 nm was because of TPA^•+^ radical formation. It had a long lifetime of 67.3 μs [Fig. 3g, j, Supplementary Table 6]. A new absorption peak at 430 nm had a short lifetime along with the redshift of the TPA^•+^ absorption peak. The 430-nm peak could be attributed to the photogenerated hole-trap state, which exhibited a high oxidation ability. This result was consistent with the degradation of pollutants via •OH radicals. This also occurred for NH_2_-UiO-66/CdS and Pt@NH_2_-UiO-66/CdS in Fig. 3h, i, respectively. The increasing redshift at 600 nm was from the absorption of TPA for TPA^•+^ cation radical formation, and the absorption of the hole-transfer-transition band-trap state with a short lifetime of nanoseconds. In particular, the lifetime of the TPA^•+^ cation radicals in Pt@NH_2_-UiO-66/CdS was 74.6 μs longer than that of pure NH_2_-UiO-66 and NH_2_-UiO-66/CdS. This revealed hole transfer from NH_2_-UiO-66 to CdS via a trap state. In addition, the kinetic profiles of the new 430-nm absorption of the trap state are shown in Fig. 3k. The 1.16-μs decay component (Supplementary Table 5) could be assigned to the lifetime of the fast hole-transfer via the mediated hole trap. The results demonstrated that efficient hole transfer from the trap state in NH_2_-UiO-66 strongly supports high HER photocatalytic activity.

### The HER mechanism

**Fig. 4:**
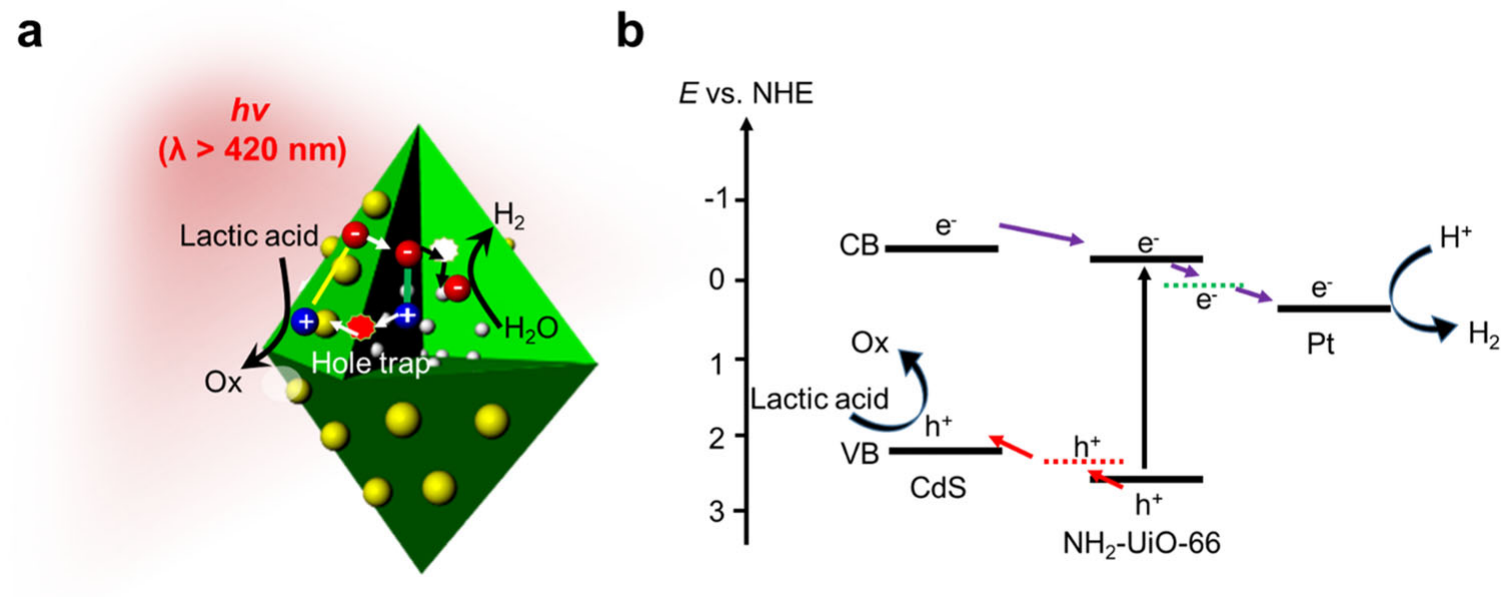
Mechanism of photocatalytic HER. **a** Mechanism of photoinduced carrier dynamics for Pt@NH_2_-UiO-66/CdS, with a hole-trap transfer pathway for enhanced HER photocatalytic activity. **b** Band diagram of photocatalytic processes in Pt@NH_2_-UiO-66/CdS.

The photoinduced carrier dynamics for photocatalytic HER by Pt@NH_2_-UiO-66/CdS HNC are illustrated in Fig. 4. When visible-light irradiated the Pt@NH_2_-UiO-66/CdS, both phases were excited, the electron from the CdS conduction band was transferred to the conduction band of NH_2_-UiO-66, and the electron was trapped. Then it was transferred to the Pt surface for HER under visible-light irradiation. The photoinduced hole in NH_2_-UiO-66 was extracted from the trapped state and transferred to the CdS valence band. Then it migrated to the surface, where it was oxidized by the sacrificial reagent. Thus, the TAS and quenching experiments using hole tracers strongly supported the mechanism of a photogenerated hole-transfer band-trap and spatial charge separation for photocatalytic HER.

## Conclusions

We reported a hole-trap transfer pathway in MOF-based ternary HER photocatalysts with spatial-separation structures. The Pt@NH_2_-UiO-66/CdS photocatalyst separated photogenerated electrons and holes by combining Pt NPs and CdS NPs, which greatly prolonged the lifetime of the hole-trap-mediated pathway and improved the HER photocatalytic efficiency. This work provides a deeper understanding of electron and hole transfer in co-catalyst-NH_2_-UiO-66-semiconductor ternary composites with spatial-separation structures. Considering their synergistic enhancement of the photocatalytic activity, the results highlight the benefits of fabricating these structures and also advance the development of highly efficient photocatalytic composites.

## Methods

### Synthesis of NH_2_-UiO-66

The procedures for synthesizing NH_2_-UiO-66 were reported previously^21^. Briefly, a mixture of 5 mL of a *N*,*N*-dimethyl formamide (DMF) solution of ZrCl_4_ (18.64 mg) and 5 mL of a DMF solution of 2-amino-1,4-benzenedicarboxylic acid (NH_2_-BDC) (14.5 mg) were mixed in a beaker. Then, 1.2 mL of acetic acid was added to the solution and transferred to a 50-mL Teflon-lined stainless-steel autoclave at 120 °C for 24 h. The product was purified via centrifugation and washed with ethanol and hexane. The NH_2_-UiO-66 was dried overnight at 60 °C under vacuum.

### Synthesis of Pt@NH_2_-UiO-66

The mixture containing ZrCl_4_ (20 mM, 10 mL) and NH_2_-BDC (20 mM, 10 mL) was shaken to form a homogeneous solution. Then, a Pt NP solution (70 μL, 30 mg mL^−1^, ~2.1 mg Pt) and acetic acid (2.74 mL) were added. After sonication for 10 min, the mixed solution was transferred to a Teflon-lined stainless-steel autoclave at 120 °Cfor 24 h. Finally, the products were collected by centrifugation and dried overnight at 60 °C under vacuum.

### Synthesis of Pt@NH_2_-UiO-66/CdS

Typically, 24.3 mg of Cd(CH_3_COO)_2_·2H_2_O was dissolved in 10 mL of ethanol forming a homogeneous solution. Then, 40 mg of Pt@NH_2_-UiO-66 was added to the solution that was then sonicated for 10 min. The suspension was heated to 80 °C at 6 °C min^−1^. At this point, 10 mL of an aqueous solution of thioacetamide (6.9 mg) was slowly injected into the flask with the rate of 0.3 mL min^−1^. It was kept at 80 °C for another 30 min. The precipitates were filtered and washed with water and ethanol several times. Finally, the product was dried at 60 °C under vacuum for 8 h.

### Characterization

TEM characterization was performed with a HT7820 (Hitachi, Japan) electron microscope at an acceleration voltage of 120 kV. High-resolution TEM, high-angle annular dark-field scanning TEM, and energy-dispersive spectrometry mapping were performed with a TalosF200x (FEI, USA, equipped with Super-X EDS) electron microscope at an acceleration voltage of 200 kV. An X-ray diffractometer (RigakuD/MAX-2000) with Cu Kα radiation (1.5406 Å) at 40 kV and 30 mA was used to record powder X-ray diffraction patterns. The patterns were collected over a 2θ range of 3°–80° at a scanning speed of 5° min^−1^. Optical absorbances of all samples were acquired with a Shimadzu UV-1900i spectrophotometer. PL spectra and time-resolved PL spectra were performed on a Hitachi F-77800 fluorescence spectrophotometer. The room temperature PL spectra were recorded with an excitation wavelength of 380 nm. The Pt content was determined via ICP-OES (Vista-MPX, Varian). Electron-paramagnetic-resonance spectra were acquired with a JEOL FA300 spectrometer with a 9.05-GHz magnetic field modulation at a microwave power of 0.998 mW. 5,5-dimethyl-1-pyrroline N-oxide (DMPO) was used as the spin-trapping reagent. Catalyst (5 mg) was suspended in the mixture of water (500 μL) and DMPO (50 μL) for the detection of •OH radicals. After ultrasonication, the detection of •OH in a N_2_ atmosphere was performed under irradiation with a 300-W Xe lamp with a 420-nm filter for 5 min. Time-resolved fluorescence decay spectra were acquired with an EI FLS-1000 fluorescence spectrometer.

### Photocatalytic activity for HER

For photocatalytic HER, the photocatalyst (5 mg) was dispersed in 25 mL of acetonitrile and 3 mL of deionized water with 3 mL of lactic acid. Then the suspension was stirred in a photocatalytic reactor and purged with N_2_ for 30 min to remove dissolved oxygen, followed by 300-W Xe light irradiation with a UV cutoff filter (>420 nm). Gas chromatography (Shimadzu GC-2014, N_2_ as a carrier gas) using a thermal conductivity detector was used to measure the HER.

The apparent quantum yield (AQY) in the above photocatalytic reaction conditions was determined. The excitation light was regulated by a bandpass filter. The intensity of the light was measured with a power meter. The AQY was calculated according to the following equation:

$${{{{{\rm{AQY}}}}}} 	=\frac{{{{{{\rm{number}}}}}}\,{{{{{\rm{of}}}}}}\,{{{{{\rm{reacted}}}}}}\,{{{{{\rm{electrons}}}}}}}{{{{{{\rm{number}}}}}}\,{{{{{\rm{of}}}}}}\,{{{{{\rm{incident}}}}}}\,{{{{{\rm{photons}}}}}}}\times 100 \% \\ 	 \, =\frac{{{{{{\rm{number}}}}}}\,{{{{{\rm{of}}}}}}\,{{{{{\rm{evolved}}}}}}\,{{{{{{\rm{H}}}}}}}_{2}\,{{{{{\rm{molecules}}}}}}\,\times \,2}{{{{{{\rm{number}}}}}}\,{{{{{\rm{of}}}}}}\,{{{{{\rm{incident}}}}}}\,{{{{{\rm{photons}}}}}}}\times 100 \%$$

## Supplementary information

Supplementary Information

## Data Availability

The datasets within the article and Supplementary Information are available from the authors upon request.
