# Supplementary material for: Photogenerated hole traps in metal-organic-framework photocatalysts for visible-light-driven hydrogen evolution
Source: Commun Chem. 2022 Aug 6;5:93. doi: 10.1038/s42004-022-00713-4 (PMC9814188; doi:10.1038/s42004-022-00713-4)
Supplement: Supplementary file 1 — Supplementary Information [file 42004_2022_713_MOESM1_ESM.pdf]

Supplementary Information

Photogenerated hole traps in metal-organic-framework photocatalysts for visible-light-driven hydrogen evolution

Zichao Lian★, Zhao Li, Fan Wu, Yueqi Zhong, Yunni Liu, Wenchao Wang, Jiangzhi Zi, Weiwei Yang

School of Materials and Chemistry, University of Shanghai for Science and Technology, Shanghai 200093, P. R. China

★Corresponding author: zichaolian@usst.edu.cn

Supplementary Methods

Synthesis of Pt nanoparticles (NPs)

Platinum (Pt) NPs were synthesized according to the previous reported method[1]. First, 6.214 mL chloroplatinic acid hexahydrate (H_2PtCl_4) solution (1 g/100 mL) was dissolved in 14 mL deionized water to form solution A. 44.3 mg of polyvinylpyrrolidone (PVP, Mw = 58,000) were dissolved in 60 mL methanol to form solution B. Then, 20 mL of solution A was dropwise into solution B under vigorously stirring. After finishing the procedures, the mixed solution was heated to 73 °C and refluxed for about 3 h. After that, the solvents were removed through the rotary evaporation method. The product of Pt NPs were collected by centrifugation, washed three times by anhydrous acetone and chloroform, and then dried at 60 °C for 8 h under vacuum. Finally, the obtained product was dissolved into 2 mL of N,N-dimethylformamide (DMF) at a concentration of ~ 30 mg mL^{-1}.

Synthesis of NH_2-UiO-66/CdS

Typically, 24.3 mg of $Cd(CH_3COO)_2 \cdot 2H_2O$ was dissolved in 10 mL of ethanol forming a homogeneous solution. Then, 40 mg of NH_2-UiO-66 was added to the solution and then they were sonicated for 10 min. The suspension was heated to 80 °C at 6°C min^{-1}. At this point, 10 mL of aqueous solution of thioacetamide (TAA, 6.9 mg) was slowly injected into the flask by dropwise. After finishing the process, it kept at 80 °C for another 30 min. The precipitates were purified and washed with hexane and ethanol several times. Finally, the product was dried at 60 °C under vacuum for 12 h.

Synthesis of Pt/NH_2-UiO-66

The synthesized NH_2-UiO-66 (58.5 mg) was dissolved in 20 mL DMF. The 4.7 μL Pt NPs DMF solution was added to the solution. The solution was ultrasoniced at room temperature for 8 h. The precipitation was collected by centrifugation, washed with ethanol for three times, and then placed overnight in a vacuum drying oven at 60 °C for 8 h.

Synthesis of Pt/NH_2-UiO-66/CdS

Typically, 24.3 mg of $Cd(CH_3COO)_2 \cdot 2H_2O$ was dissolved in 10 mL of ethanol forming a homogeneous solution. Then, 40 mg of Pt/NH_2-UiO-66 was added to the solution and then they were sonicated for 10 min. The suspension was heated to 80 °C at 6°C min^{-1}. At this point, 10 mL of aqueous solution of thioacetamide (TAA, 6.9 mg) was slowly injected into the flask by dropwise. After finishing the process, it kept at 80 °C for another 30 min. The precipitates were purified and washed with hexane and ethanol several times. Finally, the product was dried at 60 °C under vacuum for 8 h.

Synthesis of ~10 nm zb-CdS NPs

The mixture of $CdCl_2$ (0.25 mmol, 46 mg), OAc (2.5 mmol, 800 μL) and sulfur (0.25 mmol, 8 mg)/OAm (2.5 mmol, 825 μL) solution were added to di-n-octylether (10 mL) in a three-necked flask. The mixture was stirred and heated at 220 °C for 10 min under a nitrogen atmosphere. The resulting product was purified with ethanol and redispersed in hexane.

Photoelectrochemical (PEC) measurements

The PEC measurements were performed in 0.5 M Na_2SO_4 using a standard three-electrode cell with a Pt foil as the counter electrode, Ag/AgCl as the reference electrode, and the photoanodes as the working electrode, respectively. The potential vs. NHE (normal hydrogen electrode) was calculated using the following equation (Supplementary Equation 1):

$$E_{\text{vs.NHE}} = E_{\text{vs.Ag/AgCl}} + 0.1976 + 0.059 \times \text{pH} \qquad (1)$$

The as-synthesized samples (3 mg) were added into 10 μL Nafion and 0.5 mL ethanol mixed solution, and the photoanodes were prepared by dropping the suspension (150 μL) onto the surface of FTO. All the working electrodes were dried at room temperature. The photocurrent–time curves were obtained under visible light irradiation ($\lambda > 420$ nm, power density: 51.3 mW cm^{-2}) at 0.5 V (vs. RHE). The illuminated area of the working electrode was about 1 cm^2. The Mott-Schottky plots were obtained at a fixed frequency of 1 KHz to determine the flat-band potential.

Transient absorption (TA) measurements

Femtosecond (fs) TA experiments were performed with a commercial Ti/Sapphire regenerative amplifier laser system (Legend Elite-1K-HE; pulse width, 35 fs; pulse energy, 7 mJ per pulse; repetition rate, 1 kHz; 800 nm) and an automated data acquisition TA spectrometer (Helios Fire, Ultrafast Systems). Part of the fundamental (2.0 W of the amplified 800 nm output from the TOPAS, Coherent) was used for a second harmonic BBO crystal to obtain 400 nm as a pump, and the probe pulse was obtained by using amplified 800 nm output (ca. 100 mW) focused on a Ti/Sapphire crystal to generate a white-light continuum over 420–780 nm. The pump and probe beams were focused onto the sample for temporal and spatial overlap. A cell with a 2-mm optical path length was used; a stir bar kept the sample fresh and uniform. The instrument response function of this system was 100 fs.

Nanosecond to microsencond time-resolved TA spectra were acquired with a nanosecond flash photolysis setup in a Edinburgh LP920 spectrometer (Edinburgh Instruments Ltd.), combined with a compact Q-switched Nd:YAG laser (Q-smart 850, Quantel, France). The probe was a 150-W pulsed xenon arc lamp for kinetic and spectral measurements from multi-ns up to 1 ms. Photolysis of the ample was achieved with single-flash laser excitation at 355 nm (1 Hz, 10 mJ/pulse, 50 mm^2 spot area, fwhm ≈ 7 ns). The analyzing light was from a 450-W pulsed xenon lamp. The transient signals were recorded with a single detector (PMT R928P) and an oscilloscope for kinetic traces and an intensified charge-coupled device for time-resolved spectra. Data were analyzed with LP900 software. Samples with an optical absorbance of 0.6 in DMF solution at 355 nm were measured. The decay curves were fitted with biexponential functions.

Supplementary Figures and Tables

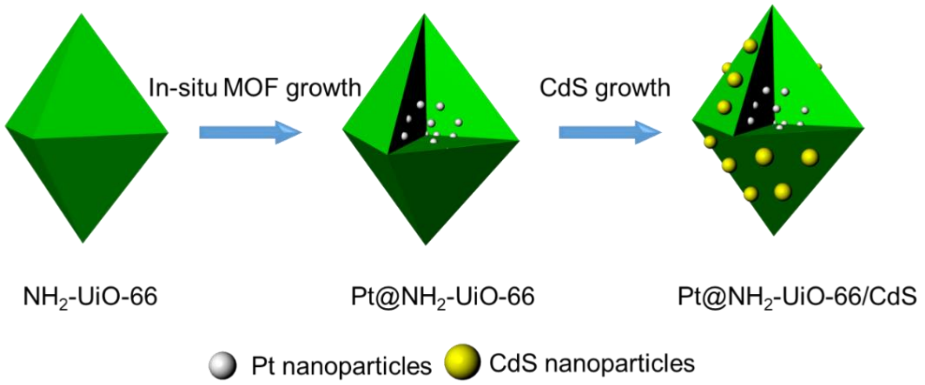

Supplementary Figure 1. Illustrated scheme of synthetic procedures. Synthetic processes of Pt@NH_2-UiO-66/CdS composites.

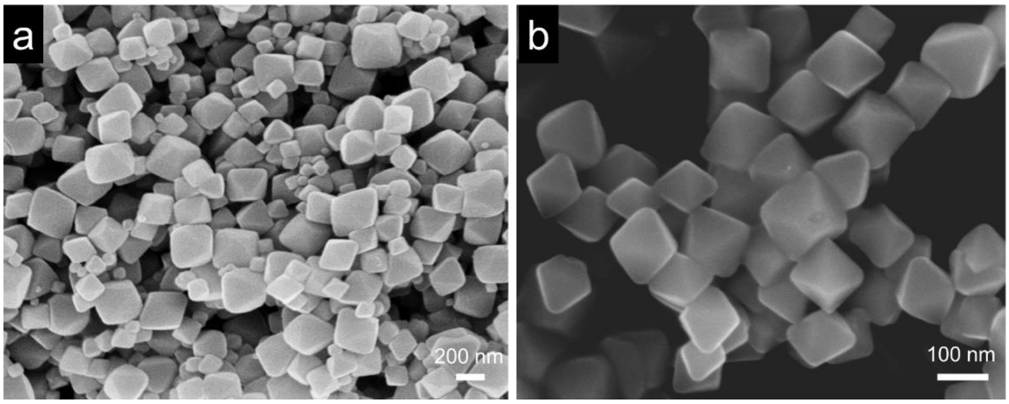

Supplementary Figure 2. Charaterization of materials. a, b, SEM images of **a** NH_2-UiO-66 and **b** Pt@ NH_2-UiO-66.

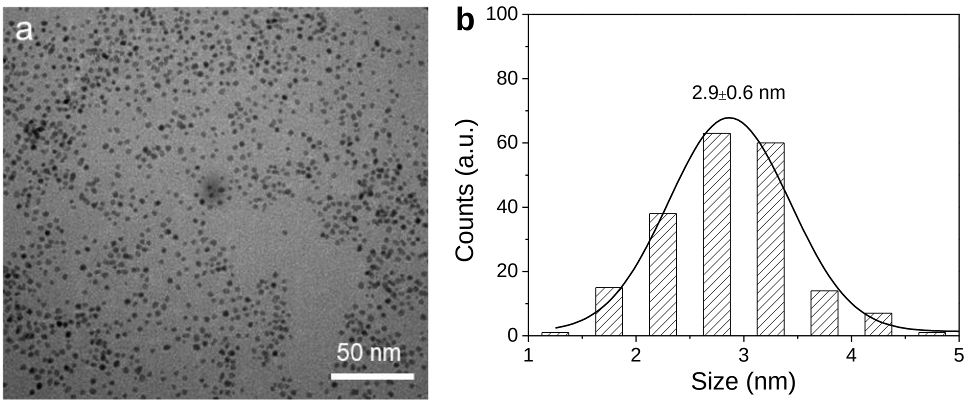

Supplementary Figure 3. Charaterization of Pt NPs. a, TEM image and **b,** scheme of size distribution of Pt NPs (2.9±0.6 nm).

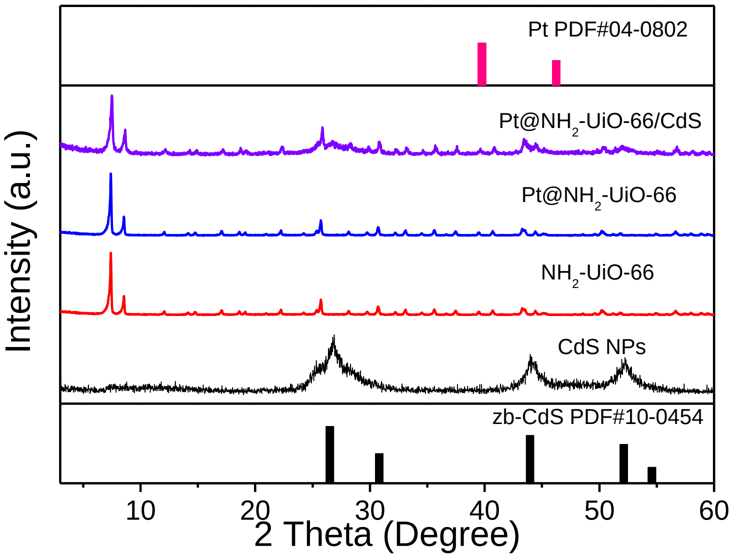

Supplementary Figure 4. Charaterization of materials. XRD patterns of CdS NPs, NH_2-UiO-66, Pt@NH_2-UiO-66 and Pt@NH_2-UiO-66/CdS.

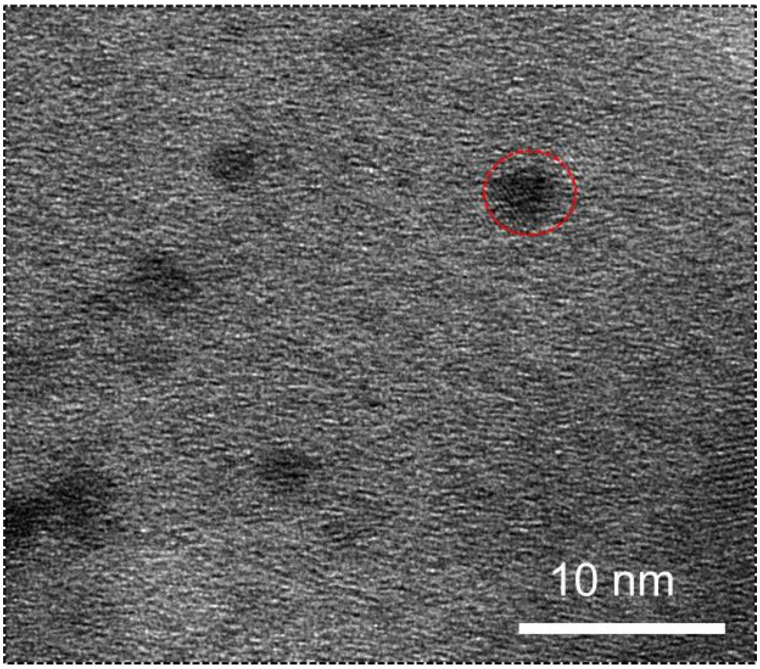

Supplementary Figure 5. High-resolution TEM image of a single Pt@NH_2-UiO-66/CdS with the highlight of the Pt by the red dashed circle. The lattice fringe could be clearly observed corresponding to the lattice plane of (200).

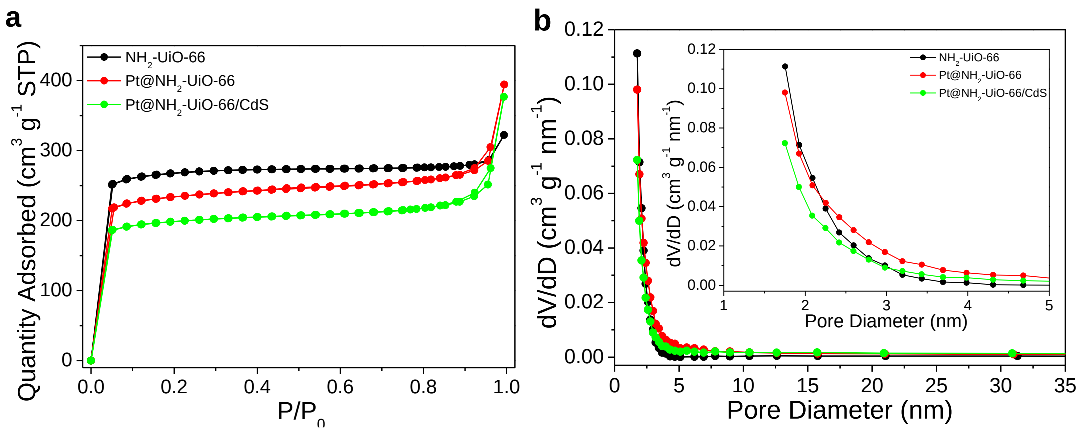

Supplementary Figure 6. Charaterization of materials. a, N_2-sorption isotherms and **b**, the pore diameter distributions of NH_2-UiO-66, Pt@NH_2-UiO-66 and Pt@NH_2-UiO-66/CdS samples. Inset of b is the pore diameter distribution at the range of 1.0-5.0 nm.

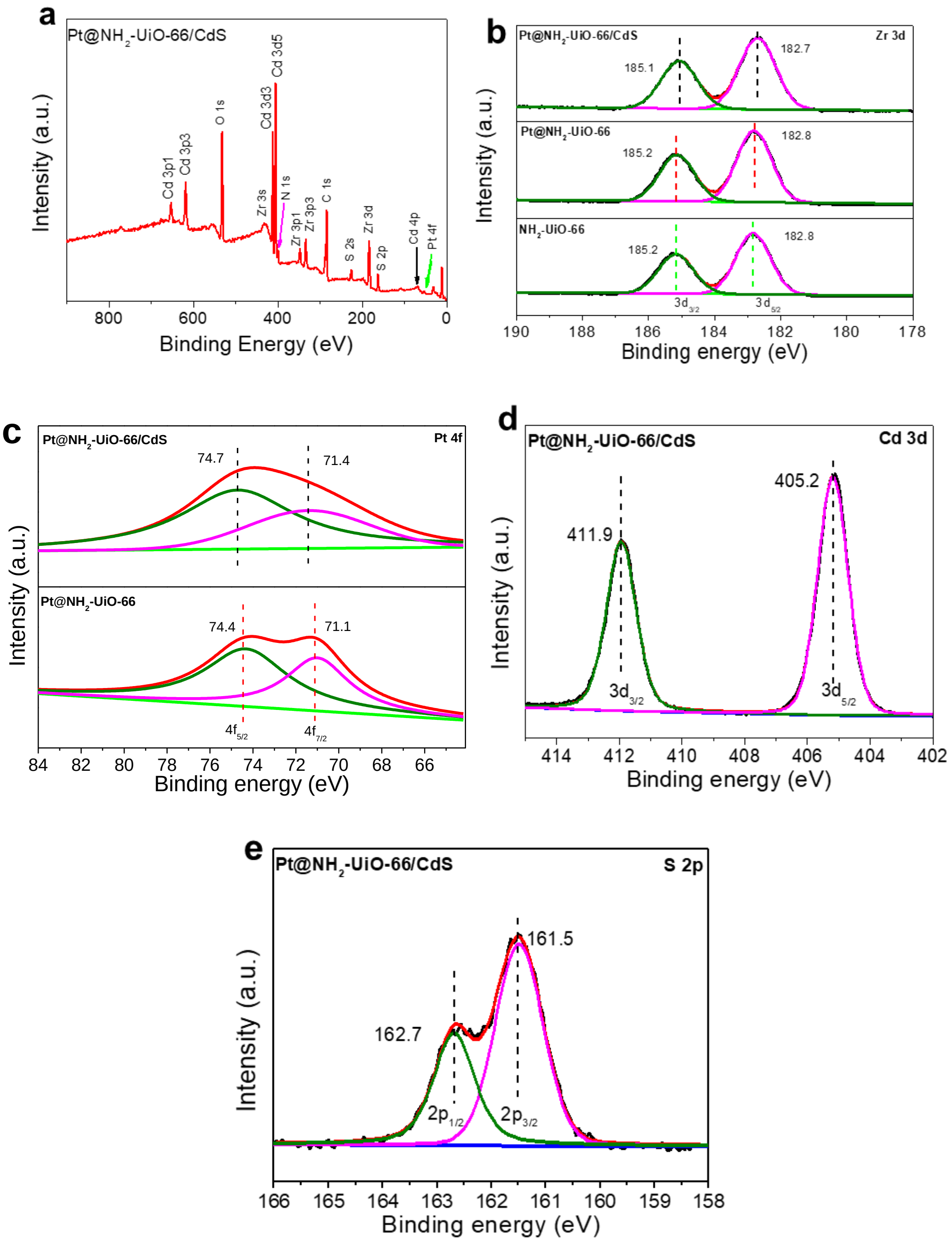

Supplementary Figure 7. XPS analysis. XPS spectra of the NH_2-UiO-66, Pt@NH_2-UiO-66 and Pt@NH_2-UiO-66/CdS: **a** survey spectrum and high-resolution; **b** Zr 3d; **c** Pt 4f; **d** Cd 3d and **e** S 2p spectra.

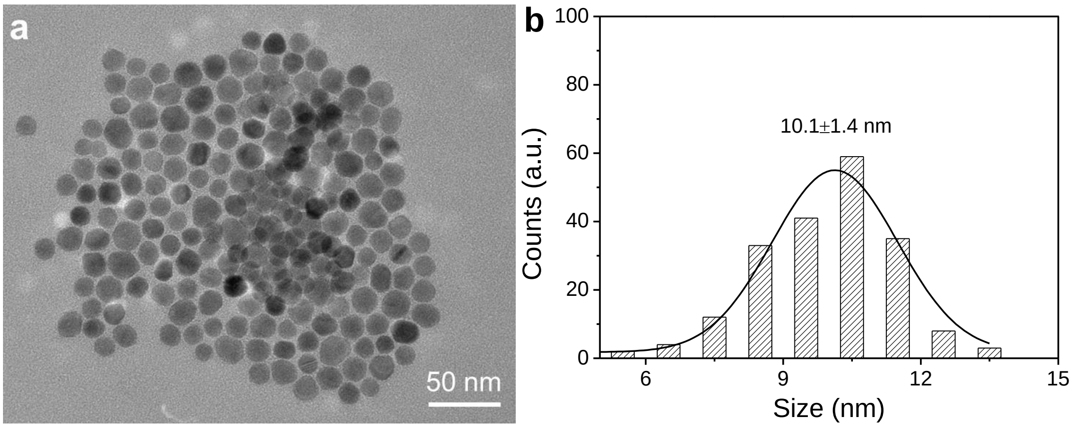

Supplementary Figure 8. Charaterization of materials. a, TEM image of a CdS NPs and **b,** scheme of size distribution of CdS NPs (10.1±1.4 nm).

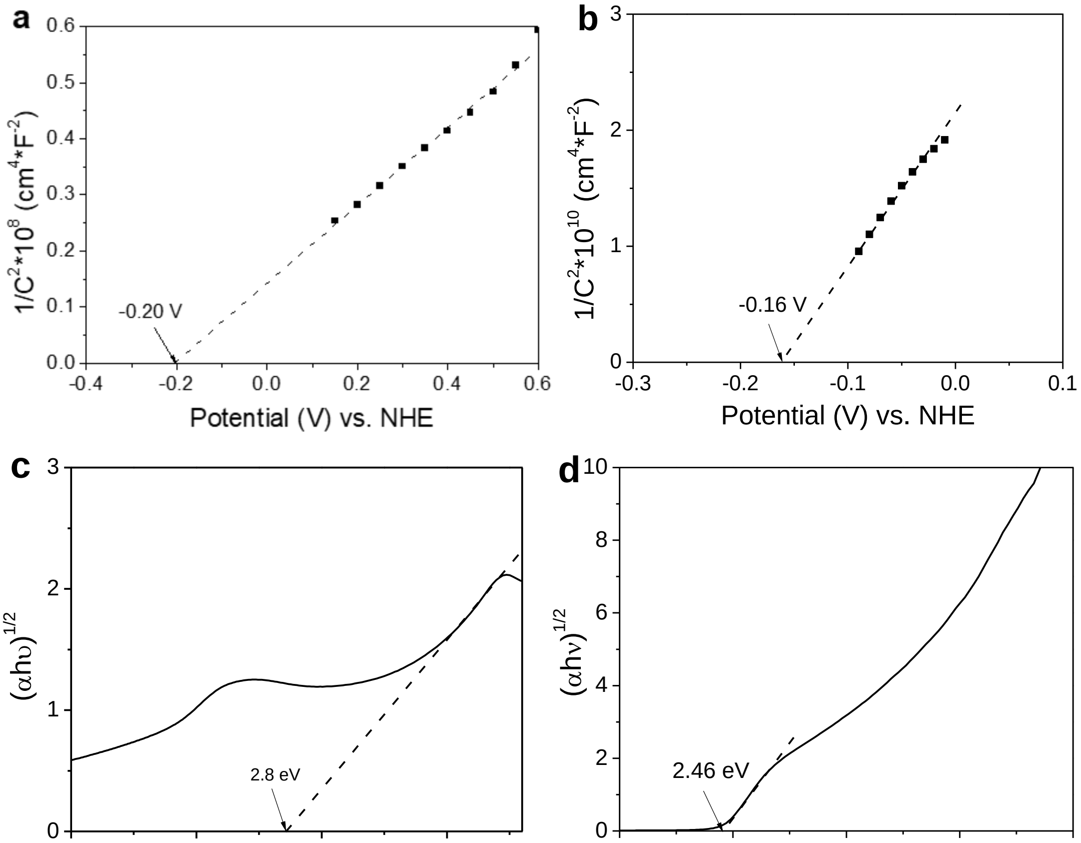

Supplementary Figure 9. Electrochemical testing and optical properties. a, **b,** Mott–Schottky plots of **a** NH_2-UiO-66 and **b** CdS NPs. **c, d,** Tauc plots to estimate bandgaps of NH_2-UiO-66 and CdS NPs. The flat band position (V_{fb}) of NH_2-UiO-66 is about −0.20 V vs NHE, approximate to the CB[2]. According to the comprehensive consideration of the relationship between the Fermi level and CB of CdS, the difference value of 0.2 eV was taken here[3].

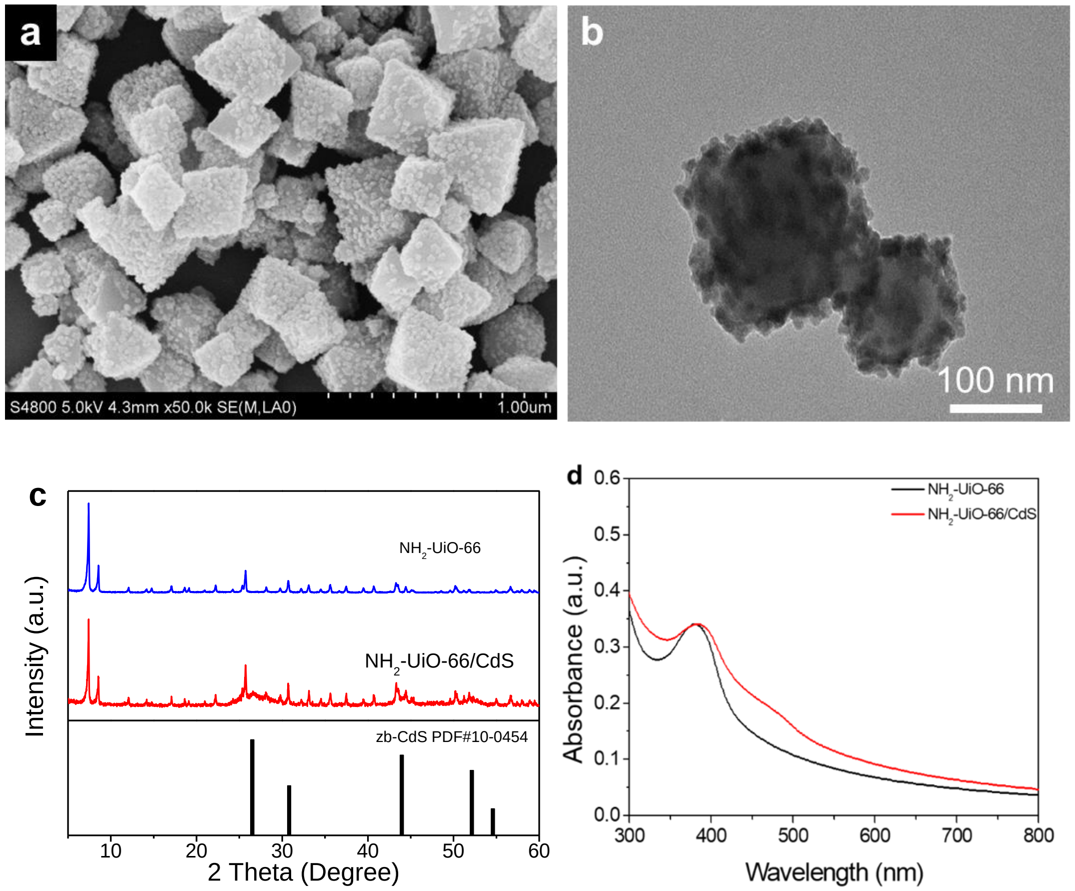

Supplementary Figure 10. Charaterization of NH_2-UiO-66/CdS. a, b, SEM **a** and TEM **b** image of NH_2-UiO-66/CdS. **c, d,** XRD patterns **c** and UV−Vis absorption spectra **d** of NH_2-UiO-66/CdS and NH_2-UiO-66, which was regared as a comparision.

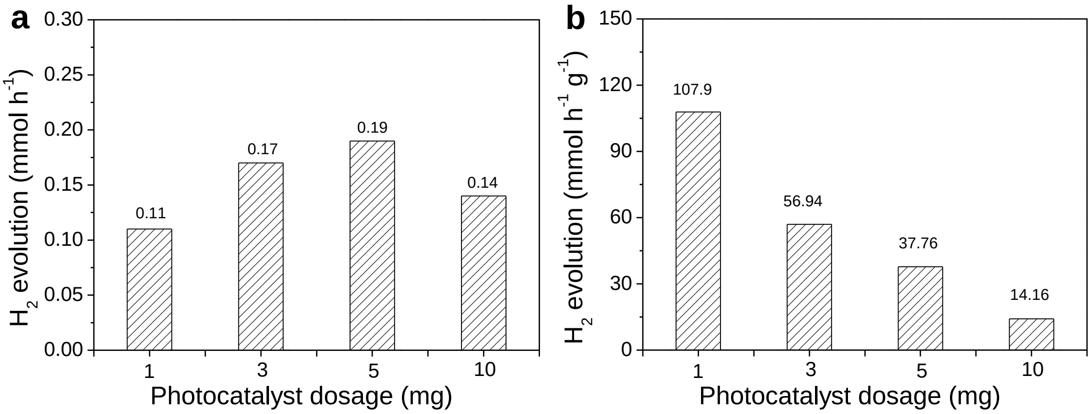

Supplementary Figure 11. Photocatalytic activity of different amounts of photocatalyst. a, The amount of the hydrogen evolution Pt@NH_2-UiO-66/CdS with different dosages; **b,** The effect of photocatalyst dosage on hydrogen evolution rate of Pt@NH_2-UiO-66/CdS Effect of photocatalyst dosage on hydrogen evolution rate (photocatalyst: Pt@NH_2-UiO-66/CdS; photocatalyst loading: 1,

3, 5 and 10 mg; reaction time: 1 h; light source: > 420 nm filter using 300 W Xe lamp)

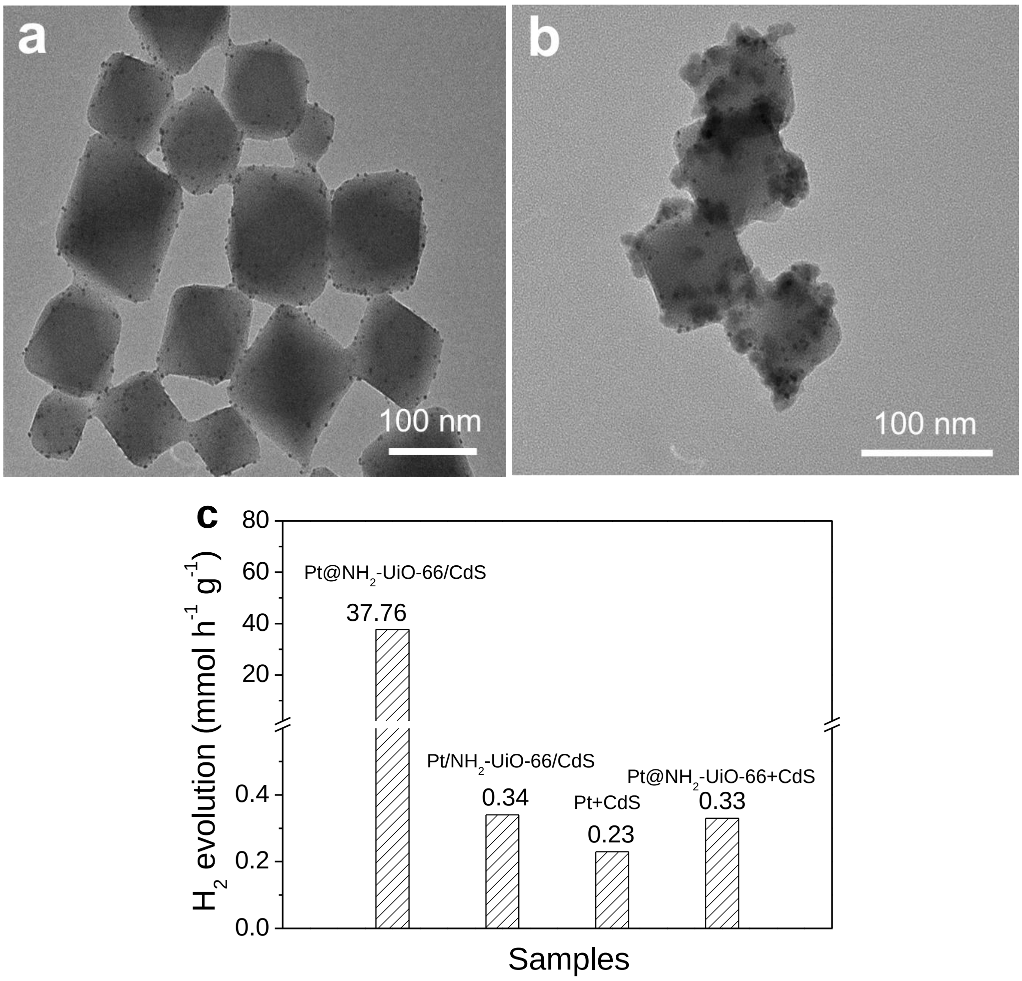

Supplementary Figure 12. Charaterization of Pt/NH_2-UiO-66/CdS and photocatalytic activity. **a, b,** TEM images of **a** Pt/NH_2-UiO-66 and **b** Pt/NH_2-UiO-66/CdS. **c,** Photocatalytic activity for HER using different physical mixtures of materials normlized by Pt contents for comparision.

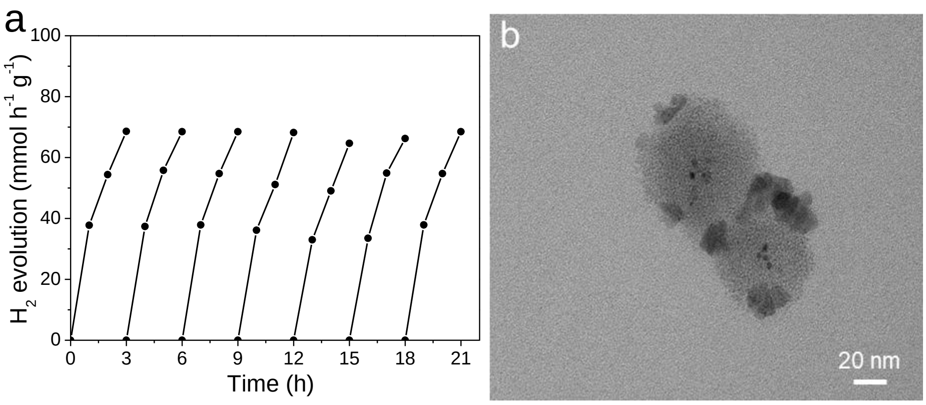

Supplementary Figure 13. Photocatalytic activity and Charaterization of NH_2-UiO-66/CdS after cycling experiments. a, Recyclability testing of Pt@NH_2-UiO-66/CdS for the HER in an acetonitrile-aqueous solution with hole scavengers (latic acid). **b,**TEM image of the Pt@NH_2-UiO-66/CdS before and after the photocatalytic HER.

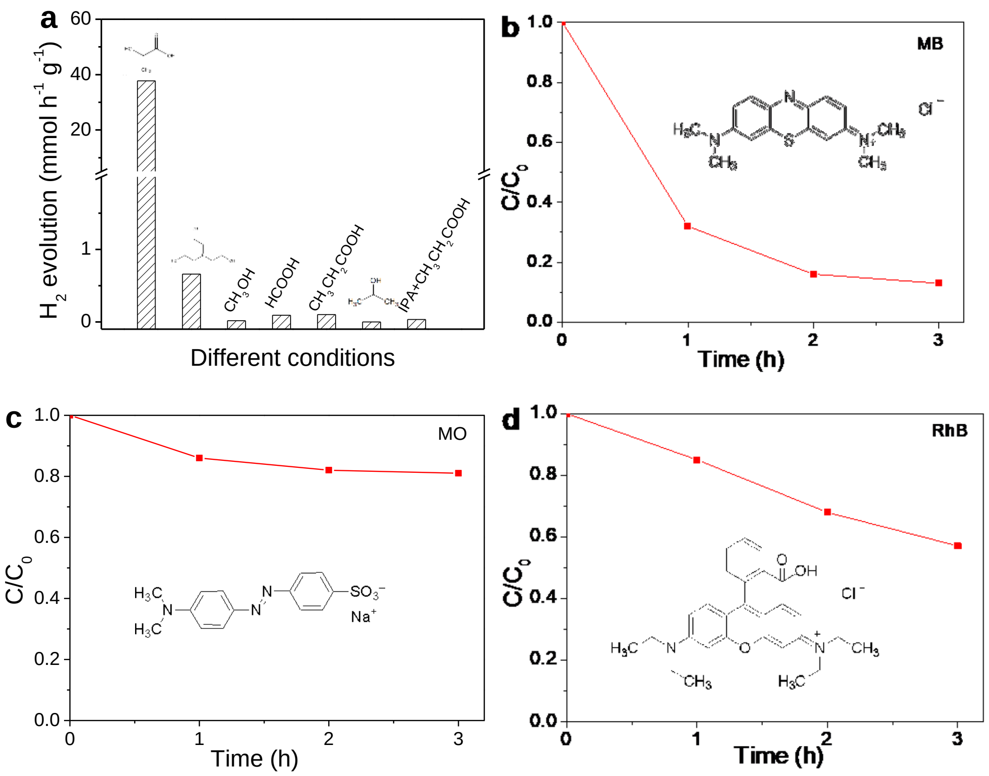

Supplementary Figure 14. Photocatalytic activity using different sacrificial agents. a, photocatalytic activity for HER of Pt@NH_2-UiO-66/CdS uisng different hole sacrificial agents. **b-d,** Degradation of different large molecules using Pt@NH_2-UiO-66/CdS, but the amount of hydrogen evolution was not observed due to the limited detection of the instrument. IPA: isopropanol.

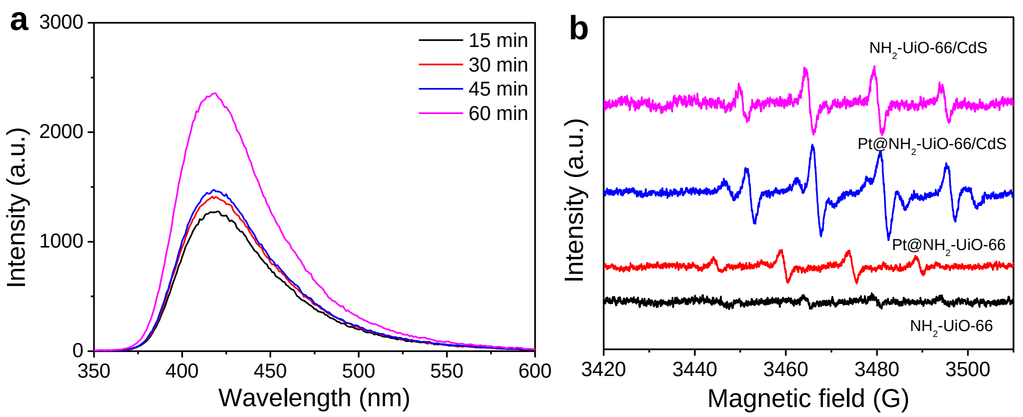

Supplementary Figure 15. The experiments of capturing the •OH radicals. a, liquid fluorescent spectrum of trapping •OH in 0.05 mol L^{-1} terephthalic acid and 0.1 mol L^{-1} NaOH solution at different irradiation time (excitation wavelength, 312 nm; emission wavelength, 426 nm) of NH_2-UiO-66, Pt@NH_2-UiO-66, Pt@NH_2-UiO-66/CdS and NH_2-UiO-66/CdS NPs. **b,** Electron paramagnetic resonance (EPR) spectra of •OH radicals trapped by DMPO over NH_2-UiO-66, Pt@NH_2-UiO-66, Pt@NH_2-UiO-66/CdS and NH_2-UiO-66/CdS NPs samples for 5 min under visible light irradiation.

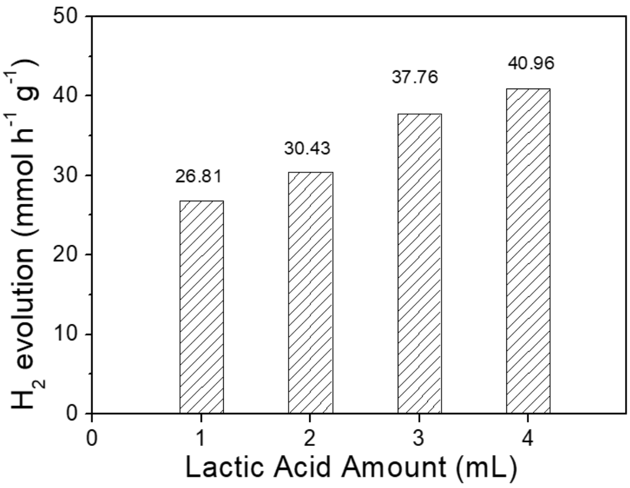

Supplementary Figure 16. Photocatalytic activity of different dosage of sacrificial agents. The photocatalytic activity of Pt@NH_2-UiO-66/CdS with different amount of lactic acid. Condition: photocatalyst loading: 5 mg; time: 1 h; light source: > 420 nm.

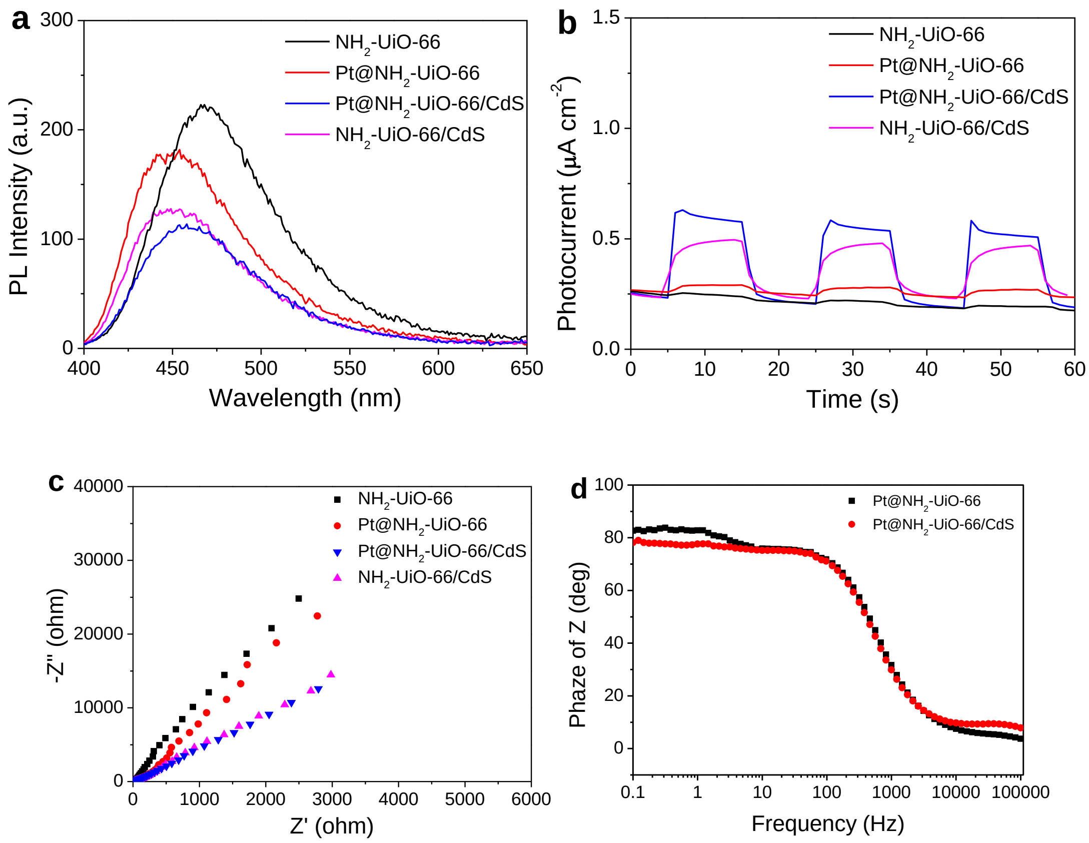

Supplementary Figure 17. Photoelectrochemical properties of materials. a, PL spectra, **b,** transient photocurrent response and **c,** Nyquist plots ofmeasured EIS spectra of NH_2-UiO-66, Pt@NH_2-UiO-66, Pt@NH_2-UiO-66/CdS and NH_2-UiO-66/CdS NPs. **d,** Bode phase plot of Pt@NH_2-UiO-66, and Pt@NH_2-UiO-66/CdS.

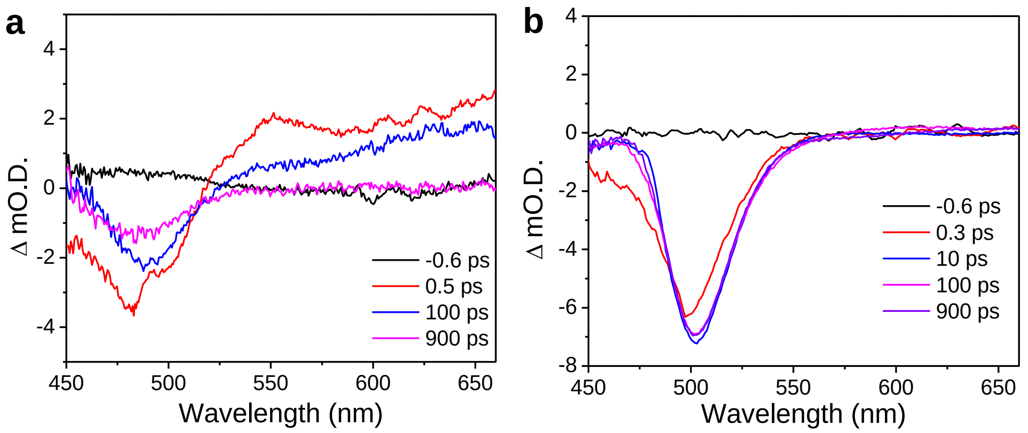

Supplementary Figure 18. Transient absorption spectra of materials. a,b, TAS of **a** NH_2-UiO-66/CdS, **b** CdS NPs with TA signal given in mOD upon 400 nm laser excitation (OD: optical density).

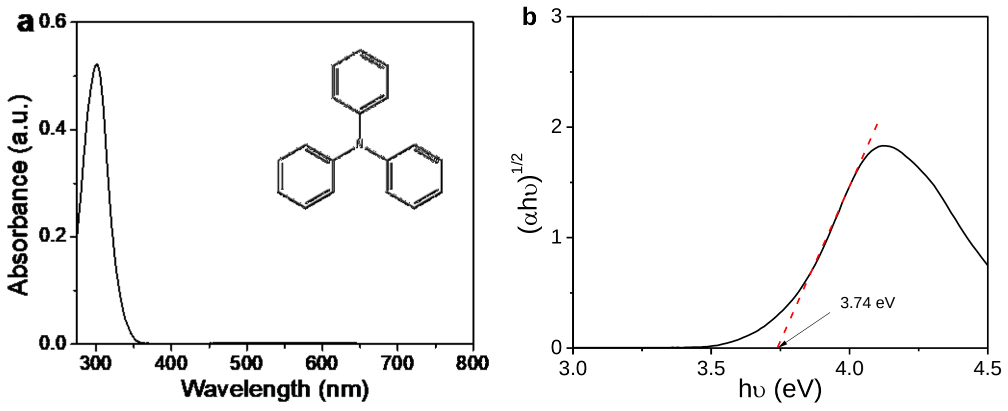

Supplementary Figure 19. Optical properties of the triphenylamine. a, UV−Vis absorption spectrum of triphenylamine (TPA). Inset: the molecule structure of TPA. **b,** Tauc plots to estimate bandgaps of TPA. The bandgaps of TPA was 3.74 eV, which could not be excited by 355 nm laser.

Supplementary Table 1. Summary of reported UiO-66 or NH_2-UiO-66 based photocatalysts of phtocatalytic activiy for HER.

Sample	Sacrificial agents	Light Source	H_2 production rate mmol g^{-1} h^{-1})	AQY (%) @wavelength	References
Pt@NH_2-UiO-66/CdS	Lactic acid	Visible light (λ > 420 nm)	37.76	40.3%@400 nm	This Wok
Pt@NH_2-UiO-66@MnO_x	TEOA	Visible light (λ > 400 nm)	1.34	-	*Adv. Mater.* **2020**, 32, 2004747
Pt@NH_2-UiO-66-100	TEOA	Full spectrum	0.38	-	*Angew. Chem. Int. Ed.* **2019**, *58*, 12175
Pt@NH_2-UiO-66	TEOA	Visible light (λ > 380 nm)	0.26	-	*Angew. Chem. Int. Ed.* **2016**, *55*, 9389
UCNPs-Pt@NH_2-UiO-66/Au	TEOA	AM 1.5G	0.28	-	*Adv. Mater.* **2018**, *30*, e1707377
$Na_{0.02}$-C_3N_4/Pt@UiO-66	TEOA	Visible light (λ > 380 nm)	0.47	-	*Chem. Eur. J.* **2018**, *24*, 18403
CdS/UiO-66	Lactic acid	Visible light (λ > 380 nm)	1.73	-	*ACS Catal.* **2018**, 8, 11615
$Cd_{0.2}Zn_{0.8}S$@UiO-66-NH_2	Na_2S/Na_2SO_3	Visible light (λ > 420 nm)	5.85	-	*Appl. Catal. B: Environ.* **2017**, 200, 448
UiOSCu-CdS/ZnS	Na_2S/Na_2SO_3	Visible light (320-780 nm)	42.55	24.6%@365 nm	*Chem. Eng. J.* **2021**, *404*, 126533
MoS_2/UiO-66/CdS	Lactic acid	Visible light (λ > 420 nm)	32.5	23.6%@420 nm	*Appl. Catal. B: Environ.* **2015**, *166*, 445

Supplementary Table 2. ICP results for Pt@NH_2-UiO-66, Pt@NH_2-UiO-66/CdS and Pt/NH_2-UiO-66/CdS samples.

Samples	Pt (wt %)
Pt@NH_2-UiO-66	0.23
Pt@NH_2-UiO-66/CdS	0.23
Pt/NH_2-UiO-66/CdS	0.22

Supplementary Table 3. The specific surface areas for NH_2-UiO-66, Pt@NH_2-UiO-66 and Pt@NH_2-UiO-66/CdS samples**.**

Samples	S_{BET} (m^2 g^{-1})	V_p (m^3 g^{-1})	D_p (nm)
NH_2-UiO-66	820	0.14	2.43
Pt@NH_2-UiO-66	722	0.32	3.38
Pt@NH_2-UiO-66/CdS	612	0.33	3.81

Supplementary Table 4. The kinetic traces of NH_2-UiO-66, Pt@ NH_2-UiO-66, NH_2-UiO-66/CdS and Pt@NH_2-UiO-66/CdS (λ_{ex} = 400 nm) were fitted by biexponential function.

Samples	Fitting parameters							
	650 nm				480 nm			
	τ_1 (ps)	A_1	τ_2 (ps)	A_2	τ_1 (ps)	A_1	τ_2 (ps)	A_2
NH_2-UiO-66	9.8	1.58	225	1.81	-	-	-	-
Pt@NH_2-UiO-66	6.2	0.80	69	0.38	-	-	-	-
NH_2-UiO-66/CdS	11.3	0.56	252	1.37	13.5	1.4	172	0.76
Pt@NH_2-UiO-66/CdS	29	0.35	120	0.21	9.3	2.6	139	3.4

Supplementary Table 5. Fitting results of the time-resolved PL spectra of NH_2-UiO-66, Pt@NH_2-UiO-66, NH_2-UiO-66/CdS, and Pt@NH_2-UiO-66/CdS samples.

Samples	τ_1 (ns)	A_1 (%)	τ_2 (ns)	A_2 (%)	τ_{Avg} (ns)
NH_2-UiO-66	0.71	57.74	3.68	42.26	1.97
Pt@NH_2-UiO-66	0.5	73.22	2.32	26.78	0.99
NH_2-UiO-66/CdS	0.46	75.83	2.9	24.17	1.05
Pt@NH_2-UiO-66/CdS	0.43	75.25	2.54	24.75	0.95

Supplementary Table 6. Fitting results of the decay profiles in the μs TAS of NH_2-UiO-66, Pt@NH_2-UiO-66, NH_2-UiO-66/CdS, and Pt@NH_2-UiO-66/CdS samples upon 355-nm laser excitation.

Samples	Fitting parameters							
	600 nm				430 nm			
	τ_1 (μs)	A_1	τ_2 (μs)	A_2	τ_1 (μs)	A_1	τ_2 (μs)	A_2
NH_2-UiO-66	21.8	0.37	67.3	0.49	21.8	0.11	82.9	0.29
NH_2-UiO-66/CdS	6.39	0.27	64.5	0.71	6.51	0.24	79.8	0.33
Pt@NH_2-UiO-66/CdS	12.6	0.44	74.6	0.38	1.16	0.60	62.9	0.49

Supplementary references

1. Zhang, W. *et al.* Exploring the fundamental roles of functionalized ligands in platinum@metal–organic framework catalysts. *ACS Appl. Mater. & Interfaces* **12**, 52660-52667 (2020).
2. Zhang, J. *et al.* Metal–organic-framework-based photocatalysts optimized by spatially separated cocatalysts for overall water splitting. *Adv. Mater.* **32,** 2004747 (2020).
3. Lian, Z. *et al.* Near infrared light induced plasmonic hot hole transfer at a nano-heterointerface. *Nat. Commun.* **9**, 2314 (2018).
